# Comprehensive Analysis of the Tegument Proteins Involved in Capsid Transport and Virion Morphogenesis of Alpha, Beta and Gamma Herpesviruses

**DOI:** 10.3390/v15102058

**Published:** 2023-10-06

**Authors:** Soumya Sucharita, Akshaya Krishnagopal, Sylvia van Drunen Littel-van den Hurk

**Affiliations:** 1 Biochemistry, Microbiology and Immunology, College of Medicine, University of Saskatchewan, Saskatoon, SK S7N 5E5, Canada; soumya.sucharita@usask.ca (S.S.); akk902@mail.usask.ca (A.K.); 2 Vaccine and Infectious Disease Organization, University of Saskatchewan, Saskatoon, SK S7N 5E3, Canada

**Keywords:** herpesviruses, tegument proteins, capsid transport, virion morphogenesis

## Abstract

Herpesviruses are enveloped and have an amorphous protein layer surrounding the capsid, which is termed the tegument. Tegument proteins perform critical functions throughout the viral life cycle. This review provides a comprehensive and comparative analysis of the roles of specific tegument proteins in capsid transport and virion morphogenesis of selected, well-studied prototypes of each of the three subfamilies of *Herpesviridae* i.e., human herpesvirus-1/herpes simplex virus-1 (*Alphaherpesvirinae*), human herpesvirus-5/cytomegalovirus (*Betaherpesvirinae*) and human herpesvirus -8/Kaposi’s sarcomavirus (*Gammaherpesvirinae*). Most of the current knowledge is based on alpha herpesviruses, in particular HSV-1. While some tegument proteins are released into the cytoplasm after virus entry, several tegument proteins remain associated with the capsid and are responsible for transport to and docking at the nucleus. After replication and capsid formation, the capsid is enveloped at the nuclear membrane, which is referred to as primary envelopment, followed by de-envelopment and release into the cytoplasm. This requires involvement of at least three tegument proteins. Subsequently, multiple interactions between tegument proteins and capsid proteins, other tegument proteins and glycoproteins are required for assembly of the virus particles and envelopment at the Golgi, with certain tegument proteins acting as the central hub for these interactions. Some redundancy in these interactions ensures appropriate morphogenesis.

## 1. Introduction

All herpesviruses belong to the family *Herpesviridae,* which includes three subfamilies, *Alphaherpesvirinae*, *Betaherpesvirinae* and *Gammaherpesvirinae*. The hosts of these herpesviruses belong to the genera *Aves, Mammalia* and *Reptilia*, although herpesviruses can also infect *Amphibia* and fish [1]. The members of the *Alphaherpesvirinae* have a short replicative cycle and cause rapid destruction of cells in culture. They establish latency in the sensory ganglia of the host, which are humans for human herpesvirus (HHV) 1 and 2 (herpes simplex virus—HSV) and HHV-3 (varicella zoster virus—VZV) or animals for bovine herpesvirus 1 (BoHV-1), swine herpesvirus-1 (pseudorabies virus—PRV) and gallid herpesvirus-2 (Marek’s disease virus—MDV). The members of the *Betaherpesvirinae* and *Gammaherpesvirinae* have a longer replicative cycle in cell culture compared to the members of the *Alphaherpesvirinae*, and establish latency in non-neuronal mononuclear cells and lymphoid cells (B and T cells), respectively. Human herpesvirus 5 (human cytomegalovirus—HCMV) and elephant endotheliotropic herpesvirus 1 (EEHV-1) are important human and animal pathogens, respectively, within the *Betaherpesvirinae* [2], and the *Gammaherpesvirinae* include HHV 4 (Epstein Barr virus—EBV), HHV-8 (Kaposi sarcoma virus—KSHV) with humans as host [3] and BoHV-4 (Bovine herpesvirus 4) infecting cattle [4,5].

HSV-1 and 2 are very common human pathogens; 67 and 13% of the worldwide population under 49 years of age has been infected with HSV-1 and HSV-2, respectively. HSV-1 and 2 have antigenic differences in their envelope proteins and cause mucocutaneous infections, which result in, often reoccurring, oral (mostly HSV-1) or genital (mostly HSV-2) lesions. These infections are transmitted from the primary site of infection (oropharyngeal or genital mucosa) to the peripheral sensory ganglia (trigeminal and sacral ganglia, respectively), where latent infection is established [6]. While reactivation may result in clinical symptoms, subclinical reactivation of HSV may occur, leading to asymptomatic shedding of the virus. HSV infection may also cause ocular herpes or encephalitis in adults, and severe encephalitis and mortality in neonates [7]. VZV primarily infects the mucosa of epithelial cells in the upper respiratory tract of humans, and ultimately infects the dermis of the skin and causes chickenpox. As a member of the *Alphaherpesvirinae*, VZV establishes latency in the sensory neurons of the cranial or dorsal root ganglia, and causes herpes zoster/shingles upon reactivation [8]. Another alphaherpesvirus, PRV, with pig as its natural host, infects the neuronal cells and causes Aujesky’s disease [9].

HCMV belongs to the *Betaherpesvirinae* and infects a wide population in early adulthood [10]. While mostly asymptomatic in immunocompetent individuals, HCMV may cause disease symptoms in individuals with a compromised immune system, in whom it can be a significant cause of morbidity and mortality [11]. For instance, HCMV may cause retinitis and blindness in immunocompromised adults. HCMV also is the leading cause of birth defects in neonates, including deafness and mental retardation [12]. HCMV establishes a primary infection in numerous cell types and is present in most organ systems [13], and it latently infects non-neuronal mononuclear cells [7]. The prevalence of HCMV is about 70% in adults. The animal betaherpesvirus EEHV-1 infects elephants, which leads to acute systemic haemorrhage in young calves [14,15]. EEHV-1 infection is widespread in North America and Europe and is responsible for about 65% of all deaths in caged Asian elephants [16].

KSHV belongs to the *Gammaherpesvirinae* and is the etiological agent of Kaposi’s sarcoma [17,18]. Kaposi’s sarcoma is the leading cause of death in AIDS patients [19,20] as it spreads from skin lesions to multiple organs like lungs and the gastrointestinal tract [20,21]. The primary site of infection is epithelial cells from which KSHV is transported to B and T-lymphocytes where it establishes latency [22,23]. BoHV-4 (*of Gammaherpesvirinae*) infects respiratory tract and ocular cells of cattle [5] and sometimes also causes genital disease and abortion [24].

All herpes virions (alpha, beta and gamma) consist of a large double-stranded DNA genome enclosed in an icosahedral proteinaceous capsid. The genome of the herpesviruses ranges from 125 kbp to 240 kbp in size, and is 32–75% GC-rich [7,25]. An amorphous protein layer surrounds the capsid, which is termed the tegument and is characteristic of herpesviruses. The tegument is further enclosed in a lipid envelope, composed of host-derived lipids and viral glycoproteins [7]. Some host proteins (annexins and tubulins) and mRNAs are also incorporated into the virions; however, these are not critical for the viral life cycle but play a role if present [26,27,28].

The focus of this review is to provide a comprehensive and comparative analysis of the functions of tegument proteins in the life cycle of select, well-studied prototypes of each of the three subfamilies of *Herpesviridae* i.e., *Alphaherpesvirinae* (HSV-1 and VZV), *Betaherpesvirinae* (HCMV) and *Gammaherpesvirinae* (KSHV). The animal viruses from the three subfamilies remain poorly characterised. We emphasise the functions of tegument proteins in the transport of capsids through the host cells and viral morphogenesis (primary and secondary envelopment), although tegument proteins play additional, diverse and important roles in the herpesvirus life cycle and infection.

## 2. Tegument Composition

In the *Herpesviridae* family, a characteristic proteinaceous layer, the tegument, forms a bridge between the lipid envelope and the nucleocapsid. The tegument is divided into outer and inner teguments based upon its interaction with the proteins of the envelope or the capsid, respectively [7,29,30]. The outer layer of the tegument is more amorphous, reflecting asymmetrical arrangements of the glycoproteins. The inner tegument has a more icosahedral symmetry because of its close association with the icosahedral capsid of the virus [31]. The tegument is an essential component of the herpesvirus structure, and the tegument proteins perform a variety of functions that are critical for the viral life cycle [32]. The dynamic process of tegument formation involves strong protein–protein interactions between the capsid proteins and the tegument proteins, between tegument proteins, and between tegument proteins and the glycoproteins or membrane-associated viral proteins of the lipid envelope [29,31,32]. The number of tegument proteins in the *Herpesviridae* ranges from 17 to 38 [33,34,35,36].

The tegument protein composition of HSV-1 has been the most extensively characterised. Proteomics studies on virions of HSV-1 indicated the presence of 24 proteins in the tegument. pU_L_7, pU_L_23 (thymidine kinase), pU_L_50 and pU_L_55 were first identified as part of the HSV-1 tegument by mass spectrometry [34]. Some proteins, such as pU_L_41 (viral host shut-off protein) and pU_L_13, have been classified as minor, but are important for virus replication [37,38], while pU_L_36, pU_L_47 (VP13/14), pU_L_48 (VP16) and pU_L_49 (VP22) are classified as major tegument proteins that are structurally important [39]. Tegument proteins pU_L_46, pU_L_47, pU_L_48 and pU_L_49 are present at 1000–2000 copies, whereas the remainder of the tegument proteins are present at fewer than 1000 copies, suggesting a significant redundancy during tegument formation [40]. The tegument is an indispensable layer of the herpesvirus, and the tegument proteins are responsible for diverse critical functions in the virus life cycle. However, pU_L_36, pU_L_37, pU_L_48 and ICP4 are the only tegument proteins that are essential for HSV-1 growth in cell culture [41]. Several tegument proteins are conserved among and across families, which suggests crucial roles of those proteins. Table 1 shows a list of the tegument proteins of HSV-1 with their functions and whether they are conserved among and across subfamilies [32,34,42].

VZV has a strong cell-associated nature, which makes isolation of virus in sufficient quantity and purity, and hence mass spectrometry analysis, difficult. The types and functions of VZV tegument proteins are predicted based on their HSV-1 homologues. Table 2 lists the VZV tegument proteins and their HSV-1 homologues. Similar to HSV-1, VZV tegument proteins play important roles in the VZV infection cycle.

PRV, an animal alphaherpesvirus, is closely related to HSV-1, HSV-2 and VZV [43,44]. PRV has a vast host range and is neuroinvasive in nature which makes it a model organism to understand the mechanism of mammalian neurons [45]. The composition of and functions performed by the PRV tegument proteins are also similar to HSV-1 [46,47,48,49,50,51,52,53,54], but remain beyond the scope of this review which focuses on human herpesviruses.

Among the *Betaherpesvirinae*, the tegument proteins of the prototype member HCMV have been characterised in the most detail. Similar to those of the *Alphaherpesvirinae*, some tegument proteins of HCMV also remain closely associated with the capsid [35,55]. The major constituent of the HCMV tegument is the phosphoprotein pp65 [56]. Most of the HCMV tegument proteins are phosphorylated, and the phosphorylation status plays an important role in regulating their functions and subcellular localisations [57]. The KSHV (*Gammaherpesvirinae*) tegument has also been studied by cryoelectron microscopy and mass spectrometry, showing similar structural arrangements as those in HSV-1 due to well-established protein–protein interactions [58]. Table 3 summarises the established homologues of HSV-1 tegument proteins in HCMV and KSHV, and the comparative functions of the proteins during different stages of the virus life cycle are described in the later sections. The tegument proteins of the animal viruses EEHV-1 (*Betaherpesvirinae*) and BoHV-4 (*Gammaherpesvirinae*) remain poorly characterised, but are likely to be conserved similar to alphaherpesviruses.

## 3. Dissociation of Tegument Proteins

Following entry of the herpesviruses into host cells via glycoprotein-receptor recognition, subsequent dissociation of the tegument layer is observed across all of the subfamilies of the *Herpesviridae.* Details of the mechanism of entry of herpesviruses are beyond the scope of this review. Tegument dissociation upon entry has been studied in depth for HSV-1 from the alpha herpesviruses.

In HSV-1, the outer tegument proteins are dissociated first, followed by the inner tegument layer. Dissociation of the tegument is required to enable the various functions of the tegument proteins [39]. The dissociated tegument proteins facilitate viral replication; for instance, pUL41 degrades the mRNAs of host transcription factors and supresses host protein production [59]. Tegument proteins are also critical to regulate apoptotic signalling [60,61,62] and to mediate suppression of host immune responses [63]. Tegument dissociation of HSV-1 is based on an energy-dependent mechanism facilitated by adenosine tri-phosphate (ATP), enzymes and magnesium ions [64]. Dissociation of the HSV-1 tegument is regulated by viral and host kinases required for phosphorylation of the tegument proteins. The requirement for kinases is based on evidence that suggests inhibition of tegument dissociation by heat inactivation of the kinases. For example, the dissociation of HSV-1 VP22 is promoted by phosphorylation by casein kinase-2 [64]. Tegument dissociation occurs in order of their requirement during viral infection and in reverse order of incorporation into the virion. In HSV-1, the first tegument protein to dissociate is VP16 [65], which travels into the nucleus to facilitate immediate early gene expression [66]. VP13/14, a minor tegument protein, dissociates next, followed by VP22. A few inner tegument proteins, like pU_L_36 and pU_L_37, exist in close proximity to the nucleocapsid, and guide the nuclear capsid towards the nuclear pore complex [64,65,67].

Based on a similar composition, a pattern similar to HSV-1 tegument dissociation is expected to be followed by all the members of the *Herpesviridae* family. However, extensive studies on the sequence and pattern of tegument protein dissociation in VZV, HCMV and KSHV have not been performed yet.

## 4. Functions of Tegument Proteins

### 4.1. Capsid Transport

After entry of the virus into the host cell, the viral capsids associate with the host microtubules to be transported within the cell and to the nucleus [68]. In HSV-1-infected cells, the tegument proteins involved in the transport of the viral capsid to the nucleus are the capsid-associated proteins pU_L_36 [69] and pU_L_37 [70]. pU_L_36 is critical as the nuclear localisation signal (NLS) of pU_L_36 navigates the capsid towards the nucleus [60]. pU_L_37 forms a complex with pU_L_36 and mimics the host transport machinery. The N-terminal region of pU_L_37 has a structural similarity to the host protein complex controlling cellular protein trafficking, and thus plays a role in the transport of the virions to the nucleus. However, it is not essential for the docking of the capsid at the nuclear pore complex [71,72]. On arrival of the capsid at the nuclear pore complex, cleavage of pU_L_36 promotes the release of the viral DNA into the nucleus [73]. ICP0 is another early tegument protein, which has E3 ubiquitin ligase activity, promoting host 26S proteasomal degradation and enabling efficient capsid delivery to the nucleus in HSV-1-infected cells [74]. Retrograde transport of the capsid during the establishment of latency in HSV-1 infected cells is facilitated by pU_S_11 [75].

Capsid transport in VZV is regulated by *orf9* through recruitment of tegument proteins IE62, IE4, IE63 and ORF47 to form a complex with microtubules. However, a detailed mechanism of capsid transport remains poorly characterised [76].

Similar to other herpesviruses, after entry into the host cell, HCMV hijacks the intracellular transport machinery to travel to the nucleus [77,78]. The transport of the capsid to the nucleus in HCMV-infected cells is predominantly microtubule-dependent [79]. pp150 is the most abundant tegument protein of HCMV and remains tightly associated with the capsid to facilitate its transport to the host nucleus by the pp150 NLS [80]. A complex formed by pU_L_47 and pU_L_48 also remains tightly associated with the viral capsid and plays a significant role in its transport to the nucleus and/or the release of the genome into the nucleus, similar to HSV-1 homologues pU_L_37 and pU_L_36, respectively [81,82]. The release of the HCMV genome into the host nucleus requires pUL47; however, the mechanism is not known [81]. Cleavage of a capsid-associated tegument protein for release of the viral genome (similar to HSV-1 pUL36) or the involvement of dynein for transport of the capsid (as in HSV-1) has not yet been identified for HCMV.

In the case of KHSV, a complex interplay between host and viral factors is responsible for the capsid transport to the nucleus. The host factors involved are predominantly microtubules [83,84,85], while dynein plays a role in the transport of the viral capsid along the microtubules [83]. However, the tegument proteins regulating the KSHV capsid transport have not been identified yet. Table 4 summarises the viral and host proteins regulating capsid transport in HSV-1-, HCMV- and KSHV-infected cells.

### 4.2. Nuclear Egress

Tegument proteins also play a major role in the egress of the virus from the nucleus, which involves crossing two nuclear membranes through envelopment and de-envelopment [86]. After the viral capsid assembly, the nucleocapsids bud at the inner nuclear membrane by disrupting the rigid nuclear lamina. The host inner nuclear membrane is acquired as an envelope by budding of the capsid into the perinuclear space by the process of primary envelopment [87,88,89]. The mechanism of nuclear egress is conserved among herpesviruses and is governed by a heterodimeric nuclear egress complex (NEC). The steps that regulate nuclear egress include formation of the complex, redistribution of the nuclear lamina and docking of the NEC at the inner nuclear membrane [90,91,92].

The most extensive and detailed studies of nuclear egress have been performed for HSV-1. HSV-1 pU_L_31 and pU_L_34 form a complex (nuclear egress complex; NEC) at the inner nuclear membrane, and promote the localisation of the nucleocapsids adjacent to the inner nuclear membrane to facilitate the budding process [93,94,95,96,97,98]. The C-terminal region of pU_L_31 forms a bridge between the nuclear membrane and the viral capsid inside the nucleus, to enable the formation of a budding vesicle by the inner nuclear membrane [99]. This complex also plays a role in disruption of the nuclear lamina to facilitate budding. The HSV-1 serine/threonine kinase pU_S_3 phosphorylates lamins and the lamin receptor emerin, resulting in destabilisation of nuclear lamina [87,88,100]. pU_S_3 also phosphorylates pU_L_31 and pU_L_34 and regulates the nuclear localisation of the pU_L_31/34 complex [88,101,102,103]. Another HSV-1 viral kinase, pU_L_13, is also likely to play a role in regulation of the localisation of the pU_L_31/34 complex, either directly or indirectly through phosphorylation of pU_S_3 [101,104].

In the de-envelopment process, the primary envelope of the virus fuses with the outer nuclear membrane for release of nucleocapsid. A host cellular component, p32, moves to the nuclear egress complex to interact with the HSV-1 pU_L_31-pU_L_34 complex to enhance the process of virion de-envelopment [105,106]. Tegument protein ICP22 or VP13/14 forms a bridge between the host p32 and the viral pU_L_31-pU_L_34 complex [107]. At the outer nuclear membrane, HSV-1 pU_S_3 phosphorylates the cytoplasmic tail of gB in the primary enveloped virion to enhance gB-mediated fusion of the primary envelope and the outer nuclear membrane [108]. Whether other viral proteins or cellular proteins are involved in de-envelopment is unclear.

In VZV-infected cells, the proteins encoded by ORF24 and ORF27 form the NEC [109]; however, the other proteins and mechanisms involved remain poorly characterised.

HCMV, similar to alphaherpesviruses, also follows the pattern of envelopment–de-envelopment for nuclear egress. The HCMV NEC is formed by pUL50 and pUL53 [92]. The NEC recruits viral kinase pU_L_97, which facilitates nuclear egress of the nucleocapsid by phosphorylation of viral pU_L_44 [110,111] and eukaryotic translation elongation factor 1δ [112]. pU_L_97 also phosphorylates the lamins causing their redistribution, and hence supports nuclear egress [113]. In KSHV, ORF67 and ORF69 form the NEC [114,115], which likely mediates phosphorylation of the lamins by recruitment of viral/host kinases to enable nuclear egress; however, there is no evidence to support this yet. Table 5 lists the proteins involved in NEC formation and kinases involved in facilitation of nuclear egress in HSV-1-, VZV-, HCMV- and KSHV-infected cells.

In summary, the mechanism of nuclear egress is conserved across herpesviruses. Two different proteins form a heterodimeric complex termed the NEC, which is responsible for recruitment of viral kinases and/or other host factors. These viral kinases phosphorylate the lamins and/or other host factors to cause their redistribution or disruption in order to facilitate nuclear egress. These viral and host factors are also responsible for docking the nucleocapsid at the inner nuclear membrane to initiate the budding process. Detailed studies of the mechanism and the specific proteins involved are yet to be performed for viruses other than HSV-1.

### 4.3. Secondary Envelopment

Secondary envelopment is the final step in the viral morphogenesis during herpesvirus maturation. Secondary envelopment involves incorporation of tegument proteins present in the cytoplasm into the virion, and eventually viral envelopment by a host-derived lipid membrane with embedded viral glycoproteins [41,116]. Molecular interactions involving viral membrane proteins, tegument proteins and capsid proteins play critical roles in the secondary envelopment and tegument incorporation [30,32]. Tegument protein pU_L_16 is conserved across *Herpesviridae* and plays a pivotal role in virion morphogenesis in the cytoplasm. Secondary envelopment has been most extensively studied for HSV-1. HSV-1 pU_L_16 binds indirectly to the capsid via interaction with pU_L_21, which is a capsid-associated tegument protein [117]. pU_L_16 also binds to pU_L_11, which is a conserved membrane-associated tegument protein. This interaction of pU_L_16 and pU_L_11 homologues is conserved among *Herpesviridae* [118]. Another HSV-1 tegument protein, VP16/pU_L_48, interacts with inner tegument proteins (pU_L_36), outer tegument proteins (pU_L_41, pU_L_46, pU_L_47 and pU_L_49) and the cytoplasmic tail of glycoproteins (gH) [31]. The tegument protein pU_L_49 interacts with membrane protein gE [32]. These interactions contribute to forming a bridge between the viral capsid and envelope during virus morphogenesis. All of these interactions play an important role in the incorporation and maintenance of the respective proteins, as well as the viral morphology [119]. A detailed description of the proteins involved and the protein–protein interactions regulating the secondary envelopment process of VZV has not yet been written. However, owing to the conserved nature of the proteins and interactions involved amongst the studied alphaherpesviruses, VZV is most likely to follow similar mechanisms.

Similar to the interaction of HSV-1 pU_L_16 and pU_L_21, HCMV pp150 encoded by the *U_L_32* gene forms an important component of the tegument network surrounding the capsid [120,121]. pp150 is phosphorylated by pU_L_96 and plays an important role in the stability of the capsid [122,123,124]. pp150 interacts with the capsid-associated proteins pp71 (*U_L_82* gene) and pp65 (*U_L_83* gene) to form a firm base for the incorporation of the outer tegument layer [125,126]. Similar to HSV-1, in HCMV, protein–protein interactions play an important role in the tegument assembly, as well as viral morphogenesis [127,128]. pU_L_24, pU_L_25 and pU_L_89 form the central hub of protein–protein interactions, but are not essential for the virion assembly and secondary envelopment [128]. Interactions between pU_L_45 and pU_L_25, pp150, pU_L_48 and pU_L_69 are also critical for the secondary envelopment of the virus particle [127,129]; however, the specific functions of the proteins and the mechanism of regulation remain unknown. Despite the existence of numerous protein–protein interactions, most of these proteins are not essential for virion morphogenesis; hence, it is possible that these overlapping interactions ensure an efficient tegumentation and secondary envelopment, as they do not depend on a single protein [43,130]. The specific interactions between tegument proteins and the outer envelope proteins have not been characterised yet; however, due to the conserved nature of many interactions, HCMV is likely to have similar interactions between tegument proteins and envelope proteins as HSV-1.

In gammaherpesviruses such as KSHV, proteins encoded by ORF33 and ORF45 are conserved and play an important role in virion morphogenesis and secondary envelopment [131]. Similar to HSV-1 and HCMV, KSHV tegument proteins also interact with the capsid protein and with other tegument proteins. The ORF64 protein serves as the hub protein as it interacts with multiple other proteins [36]. ORF64 interacts with capsid proteins ORF25, ORF26 and ORF62, as well as with other tegument proteins, including ORF11, ORF12, ORF33, ORF45, ORF63, ORF75 and ORF64 itself. These interactions play an important role in the virion assembly, as well as the final envelopment of the virus [58]. These studies indicate a role of ORF64 as a bridge between capsid proteins and the outer tegument proteins to stabilise virion morphogenesis. Interactions between KSHV tegument proteins and glycoproteins that are involved in viral morphogenesis are poorly characterised. Table 6 summarises the protein–protein interactions that play a role in the assembly of HSV-1, HCMV and KSHV.

In summary, the major role of the tegument proteins during tegument incorporation and secondary envelopment is interaction with other proteins. The tegument proteins interact with capsid proteins, other tegument proteins and some glycoproteins to facilitate virion morphogenesis, as well as secondary envelopment. Certain proteins act as the central hub for interactions, such as pUL48 in HSV-1; pU_L_24, pU_L_25, and pU_L_89 in HCMV; and ORF 64 protein in KSHV. The protein interactions are overlapping, such that the maturation of the virus becomes more efficient as there is no dependence on a single protein or interaction. Although the interactions between capsid and tegument proteins and between tegument proteins have been studied in detail, the tegument–glycoprotein interactions remain poorly characterised for HCMV and KSHV.

## 5. Cellular Proteins Involved in Capsid Transport and Virus Assembly

Apart from viral protein-protein interactions, interactions between host protein and viral proteins also play an important role in the viral morphogenesis and egress. After the entry of the herpesvirus into the host, the HSV-1 tegument proteins pUL36 and pUL37 recruit the host motor proteins, kinesin and dynein, and their co-factors to transport the capsid from the infection site and dock it onto the nucleus [28,50,53,65,67,132]. During the nuclear egress, pUL31/34 complex binds lamin A and C and mediates nuclear lamina disruption [95]. A detailed description of nuclear egress has been provided in the review by Klupp and Mettenleiter [133]. The host factor TRIM 43 mediates degradation of the centrosomal protein, pericentrin, which leads to disruption of the nuclear lamina and aids in viral nuclear egress [134]. Similar involvement of viral-host protein interactions is also present in beta and gamma herpesviruses [135,136]. Although protein-protein interactions between virus and host proteins are important for the viral spread as well as morphogenesis and maturation of the virus, this review focuses on the protein-protein interactions between the viral tegument proteins.

## 6. Discussion and Conclusions

**Figure 1:**
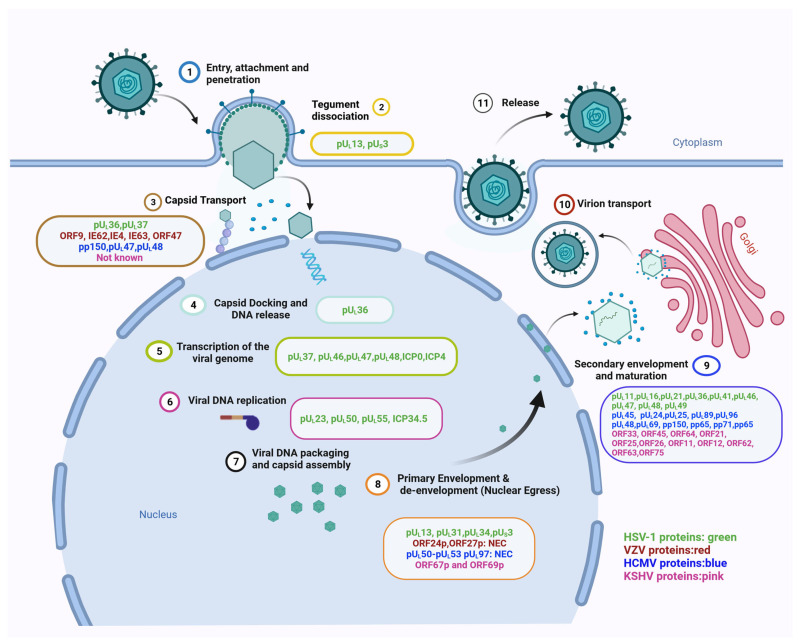
Herpesvirus life cycle highlighted with the respective tegument proteins involved in each step. Proteins of the prototypes of each of the three sub-families of Herpesviridae i.e., *Alphaherpesvirinae* (HSV-1 and VZV) are highlighted in green and red, *Betaherpesvirinae* (HCMV) in blue and *Gammaherpesvirinae* (KSHV) in pink, respectively. It is to be noted that the schematic mentions the tegument proteins involved in the HSV-1 life cycle in detail, and highlights only the known tegument proteins for VZV, HCMV and KSHV, as most of their proteins have not been characterised yet; however, due to their conserved nature, the homologous tegument proteins of VZV and HSV-1 are likely to have similar functions. (1) Virus entry of the host cell through attachment and membrane fusion; (2) Dissociation of several tegument proteins; (3) Capsid transport to nucleus. (4) Docking of the capsid onto the nuclear pore complex and release of the viral DNA; (5) Transcription of the viral genome; (6) Replication by rolling circle mechanism; (7) Viral capsid formation and viral DNA packaging; (8) Primary envelopment and de-envelopment of the capsid; (9) Secondary envelopment, which enables the virus to mature; (10) Virion transport and (11) Release of the mature virus from the cell via exocytosis after re-envelopment.

The herpesvirus tegument proteins play important roles in the capsid transport to and docking at the nucleus after virus entry, as well as the nuclear egress and primary envelopment of new capsids (Figure 1). All herpesviruses characterised thus far use the intracellular transport machinery to travel to the nucleus after entry into the host cell; several capsid-associated tegument proteins are involved in the association with the microtubules and dynein. Based on current knowledge, the mechanism of nuclear egress is conserved across herpesviruses; several tegument proteins with defined, similar functions in the modulation of the cellular structures involved in this process have been identified. During secondary envelopment, multiple tegument–tegument protein interactions, as well as interactions of tegument proteins with capsid proteins and glycoproteins, are responsible for morphogenesis of the virions, with certain tegument proteins acting as the hub for multiple protein–protein interactions. Overall, there is some redundancy in the functions of the tegument proteins, ensuring appropriate morphogenesis. Among the human herpesviruses, much of the currently available information on the tegument proteins’ functions in virion transport and morphogenesis is based on alpha herpesviruses, in particular, HSV-1. While there is some information on HCMV and KSHV, there is still much to be learned about the functions of the beta and gamma herpesvirus tegument proteins in this regard, and it cannot be assumed that homologous proteins always have similar functions. Among the many veterinary herpesviruses, the morphogenesis of PRV has been extensively studied [43,133]. However, while several functions of tegument proteins from other veterinary alpha herpesviruses, such as BHV-1, EHV-1 or MDV, in context of immune modulation or latency, have been identified [137,138,139,140,141], not much is known about their roles in virion transport or assembly.

## 7. Future Directions

More advanced technologies such as liquid chromatography-mass spectroscopy (LS-MS), high-resolution confocal microscopy and bimolecular fluorescence complementation (BiFC) assays will contribute to a more detailed elucidation of protein–protein interactions of tegument proteins. The use of Bacmids allows the rapid generation of mutant viruses, thus facilitating analysis of the functions of individual tegument proteins in assembly. Involvement of host proteins in the processes of capsid transport, nuclear egress and assembly can be more easily studied by using CRISPR/Cas systems. Since the assembly process could be a target for antiviral drugs, elucidation of additional tegument proteins that are critical in the morphogenesis of herpesviruses could lead to target identification for therapeutics.

## Figures and Tables

**Table 1:** HSV-1 tegument proteins with functions.

Gene	Tegument Protein	Essential (E) or Non-Essential (NE) for HSV-1 in Cell Culture	Function	Gene Conserved in *Herpesviridae* Subfamilies		
				Alpha	Beta	Gamma
*U_L_7*	pU_L_7	NE	Mitochondrial function regulation.	Yes	Yes	Yes
*U_L_11*	pU_L_11	NE	Secondary envelopment.	Yes	Yes	Yes
*U_L_13*	pU_L_13	NE	Protein kinase, tegument dissociation, inhibition of interferon response.	Yes	Yes	Yes
*U_L_14*	pU_L_14	NE	Regulation of apoptosis, nuclear targeting of capsids.	Yes	Yes	Yes
*U_L_16*	pU_L_16	NE	Secondary envelopment.	Yes	Yes	Yes
*U_L_21*	pU_L_21	NE	Secondary envelopment, regulation of microtubule assembly.	Yes	Yes	Yes
*U_L_23*	pU_L_23	NE	Thymidine kinase, viral DNA replication.	Yes	No	Yes
*U_L_36*	VP1/2 (pU_L_36)	E	Capsid transport, secondary envelopment and release of viral DNA.	Yes	Yes	Yes
*U_L_37*	pU_L_37	E	Capsid transport, secondary envelopment, viral transcription regulation.	Yes	Yes	Yes
*U_L_41*	pU_L_41	NE	Shuts off host translation and immune response evasion.	Yes	No	No
*U_L_46*	VP11/12(pU_L_46)	NE	Secondary envelopment, regulation of pUL48 dependent transcription.	Yes	No	No
*U_L_47*	VP13/14(pU_L_47)	NE	Secondary envelopment, regulation of pUL48 dependent transcription.	Yes	No	No
*U_L_48*	VP16(pU_L_48)	E	Secondary envelopment, regulation of viral transcription.	Yes	No	No
*U_L_49*	VP22(pU_L_49)	NE	Secondary envelopment, regulation of microtubule assembly.	Yes	No	No
*U_L_50*	pU_L_50	NE	Viral DNA replication.	Yes	No	No
*U_L_51*	pU_L_51	NE	Cell-to-cell spread.	Yes	Yes	Yes
*U_L_55*	pU_L_55	NE	Viral DNA replication.	Yes	No	No
*U_S_2*	pU_S_2	NE	Regulation of ubiquitination.	No	No	No
*U_S_3*	pU_S_3	NE	Protein kinase, primary de-envelopment, tegument dissociation, regulation of actin assembly.	Yes	No	No
*U_S_10*	pU_S_10	NE	Unknown	No	No	No
*U_S_11*	pU_S_11	NE	Host translational regulation, capsid transport.	No	No	No
	ICP34.5	NE	Host translational regulation, viral DNA replication and immune response evasion.	No	No	No
*RL2*	ICP0	NE	Virus transcription regulation.	Yes	No	No
*RS1*	ICP4	E	Virus transcription regulation.	Yes	No	No

**Table 2:** VZV tegument proteins and their HSV-1 homologues.

VZV Tegument Protein	HSV-1 Homologues	VZV Tegument Protein	HSV-1 Homologues
ORF53	pU_L_7	ORF10	VP16 (pU_L_48)
ORF49	pU_L_11	ORF9	VP22 (pU_L_49)
ORF47	pU_L_13	ORF8	pU_L_50
ORF46	pU_L_14	ORF7	pU_L_51
ORF44	pU_L_16	ORF3	pU_L_55
ORF38	pU_L_21	ORF66	pU_S_3
ORF36	pU_L_23	ORF64/69	pU_S_10
ORF22	VP1/2 (pU_L_36)	ORF61	ICP0
ORF21	pU_L_37	ORF62/71	ICP4
ORF17	pU_L_41		
ORF12	VP11/12 (pU_L_46)		
ORF11	VP13/14 (pU_L_47)		

**Table 3:** HSV-1 tegument proteins and their homologues in HCMV and KSHV.

HSV-1 Tegument Protein	HCMV Homologue	KSHV Homologue	HSV-1 Tegument Protein	HCMV Homologue	KSHV Homologue
pU_L_7	pU_L_103	ORF42	VP22 (pU_L_49)	NA	NA
pU_L_11	pU_L_99	ORF38	pU_L_50	NA	NA
pU_L_13	pU_L_97	ORF36	pU_L_51	pU_L_71	ORF55
pU_L_14	pU95	ORF34	pU_L_55	NA	NA
pU_L_16	pU_L_94	ORF33	pU_S_2	NA	NA
pU_L_21	pU_L_87	ORF24	pU_S_3	NA	NA
pU_L_23	NA	ORF21	pU_S_10	NA	NA
VP1/2 (pU_L_36)	pU_L_48	ORF64	pU_S_11	NA	NA
pU_L_37	pU_L_47	ORF63	ICP34.5	NA	NA
pU_L_41	NA	NA	ICP0	NA	NA
VP11/12 (pU_L_46)	NA	NA	ICP4	NA	NA
VP13/14 (pU_L_47)	NA	NA			
VP16 (pU_L_48)	NA	NA			

**Table 4:** Proteins involved in capsid transport of HSV-1, VZV, HCMV and KSHV.

Virus	Viral Proteins Involved in Capsid Transport	Host Proteins Involved in Capsid Transport	Mechanism of Genome Release	References
HSV-1	pU_L_36 and pU_L_37	Microtubules, dynein/dynactin complex	Cleavage of pU_L_36	[69,70,71,72]
VZV	ORF9, IE62, IE4, IE63 and ORF47	Microtubules	Not known	[76]
HCMV	pp150 and pU_L_47–pU_L_48	Microtubules	Not known	[77,78,79,80]
KSHV	Not known	Microtubules, dynein	Not known	[83,84,85]

**Table 5:** List of proteins involved in nuclear egress of capsids.

Viruses	NEC	Kinases Involved in Egress	References
HSV-1	pU_L_31-pU_L_34	pU_S_3	[86,93,94,95,96,97,98,100]
VZV	ORF24p and ORF27p	Unknown	[109]
HCMV	pU_L_50-pU_L_53	pU_L_97	[92,110,111,113]
KSHV	ORF67p and ORF69p	Unknown	[114,115]

**Table 6:** List of protein–protein interactions involved in secondary envelopment.

Virus	Protein(s) Forming the Hub for Interactions	Protein–Protein Interactions Playing a Role in Secondary Envelopment	References
HSV-1	pU_L_48	pU_L_16-pU_L_21; pU_L_16-pU_L_11; pU_L_48-pU_L_36; pU_L_48-pU_L_41; pU_L_48-pU_L_46; pU_L_48-pU_L_47; pU_L_48-pU_L_49; pU_L_48-gH; pU_L_49-gE	[31,32,117,118,119]
HCMV	pU_L_24, pU_L_25, pU_L_89	pp150-pU_L_96; pp150-pp71; pp150-pp65; pU_L_45-pU_L_25; pU_L_45-pU_L_48; pU_L_45-pU_L_69	[120,121,122,123,124,125,126,127,128,129]
KSHV	ORF64	ORF64-ORF64; ORF64-ORF11; ORF64-ORF12; ORF64-ORF21; ORF64-ORF25; ORF64-ORF26; ORF64-ORF62; ORF64-ORF33; ORF64-ORF45; ORF64-ORF63; ORF64-ORF75	[36,58,131]
